# Optimization of robotic spray painting trajectories using machine learning for improved surface quality

**DOI:** 10.1038/s41598-025-03448-z

**Published:** 2025-05-29

**Authors:** Ritesh Bhat, M. Karuppasamy, M. Maragatharajan, Anandakumar Haldorai, E. Nirmala, Nithesh Naik

**Affiliations:** 1 Department of Mechatronics Engineering, Rajalakshmi Engineering College, Thandalam, Tamil Nadu 602105 India; 2 Department of Computer Science Engineering (Cyber Security), ACS College of Engineering, Bengaluru, Karnataka 560074 India; 3 School of Computing Science and Engineering, VIT Bhopal University, Kothrikalan, Madhya Pradesh 466114 India; 4 Department of Computer Science and Engineering, Sri Eshwar College of Engineering, Coimbatore, Tamil Nadu 641202 India; 5 Department of Mechanical and Industrial Engineering, Manipal Institute of Technology, Manipal Academy of Higher Education, Manipal, Karnataka 576104 India

**Keywords:** Random forest, Regression model, Spray painting, Paint thickness, Surface roughness, Energy science and technology, Engineering, Mathematics and computing

## Abstract

The production process needs spray painting particularly within automobile manufacturing since product painting accuracy establishes product quality. The combination of hand spray techniques produces intricate designs as well as small quantity needs yet industrial robots excel at painting large industrial product orders. Taguchi Design of Experiments (DoE) is used to investigate the effect of six process variables which included spray distance along with pressure, temperature, humidity level, speed and viscosity rate. Experiments were conducted via industrial robotic spraying with subsequent statistical evaluation through ANOVA tests and regression calculations. The research shows that viscosity together with temperature stands as primary influential factors for thickness deviation, yet speed and temperature jointly determine surface roughness outcomes. The predictive model performed with substantial accuracy based on its ability to achieve R² values of 0.9224for surface roughness measurements and 0.9707 for thickness variation determination. The study offers clear guidelines for practitioners to enhance their processes to produce high-quality products and time efficiency.

## Introduction

Spray painting has been used for over a century, evolving significantly in application techniques and effectiveness. Initially performed manually using airbrushes or supplementary instruments^1^, it is still applied in highly automated industries. However, most industrial applications now rely on automated spray painting, improving quality and reducing costs. Automated linear spray systems have progressed into fully robotized paint booths, widely adopted across various industries. In robotic spray painting, robot trajectories are typically generated through manual teaching. Offline simulation aids engineers by providing accurate feedback on paint thickness for a given trajectory, allowing adjustments before physical implementation^2^. Once a forward simulation process is established, the inverse problem can be addressed, wherein optimal painting paths are derived based on desired paint thickness^3^. These trajectories must adhere to execution constraints while ensuring an acceptable coating^4^. While automation enhances quality and efficiency, surface quality remains a challenge, particularly when incorporating low-cost fillers in spray coatings. Fillers can increase surface roughness, but research shows that careful control of key parameters such as spray distance, pressure, temperature, humidity, speed, and viscosity can mitigate this issue^5^. The selection of smoothness or roughness depends on specific application requirements, demonstrating that cost-effective coatings can achieve desired surface properties. Various controllable parameters influence painting process quality, including pressure, transfer rate, viscosity, and paint temperature. Among them, spray gun distance, pressure, and speed are primary factors, while chemical and environmental properties remain constant^5^. Robotic spray painting is integral to manufacturing sectors such as automotive, aerospace, and furniture. Its applications extend to agriculture, pest control, and artistic restoration, driven by advancements in automation and control strategies^6^. As production requirements increase, conventional robot trajectory models no longer suffice, leading to the development of new spray modeling techniques and optimization algorithms for high-performance robotic painting^7^. Surface modeling is critical in trajectory planning, yet the diversity and complexity of workpieces pose challenges^8^. Various deposition models have been established, ranging from basic surface verification to advanced simulations of charged painting particles, employing projection techniques based on composite Gaussian formulations^9^. Accurate paint build-up prediction requires solving coupled non-linear differential equations, but this computational complexity hinders optimization^10,11^.

A simplified approach considers functional dependence between thickness and trajectory, relying on experimentally calibrated fit parameters^12^. Several trajectory optimization methods have been explored. Early studies developed circular painting patterns for uniform deposition on CAD models^15^. Subsequent research extended these techniques to broader surfaces, optimizing the overlap between adjacent sweeps^16^. Some methods construct painting paths using intersecting planes along the surface’s bounding box, while others incorporate post-processing for consistent overlap on curved surfaces^17^. Minimum geodesic curvature curves have also been used for trajectory generation on triangulated 2D surfaces^18^. Despite these advancements, challenges remain in optimizing paint thickness, cycle time, and material waste. Integer programming has been proposed for solving this problem, influencing trajectory optimization in thermal spraying^19^. Recent research has leveraged real-time adaptive control and machine learning to enhance deposition quality and minimize waste. Integrated sensor feedback models dynamically adjust trajectories based on surface irregularities, reducing overspray and improving uniformity on complex surfaces^20^. Deep learning models have been employed to predict optimal spray patterns for robotic sprayers, reducing computational costs while maintaining precision^21^. Studies have also analyzed the impact of material properties on deposition along predicted trajectories, demonstrating that dynamic parameter adjustments can enhance adhesion and durability^22^. AI-based systems further refine the process by automatically adjusting spray speed and angle based on real-time environmental conditions, achieving uniform multilayer coatings with reduced material waste^23^. A multi-objective optimization framework for controlling spray parameters across multiple layers ensures even distribution, anticipating layer interactions to improve overall coating quality^24^. Despite these advancements, robotic spray gun path planning remains limited to simple geometries and struggles with complex shapes. The interdependence of parameters such as pressure, distance, speed, temperature, humidity, and viscosity complicate achieving consistent paint thickness and surface smoothness. The present study focuses on these six factors due to their significant influence on paint quality and adjustability in industrial settings. The proposed method incorporates a paint projection profile without calibration parameters, using physical experiments to refine the deposition model. The objective function accounts for system dynamics during painting trajectories. An orthogonal array table and machine learning techniques, including random forest and linear regression, were applied to evaluate performance. Paint thickness and surface roughness were measured at various points, demonstrating the applicability of this approach to robotic paint guns executing linear motion commands. The results validate the effectiveness of random forest regression in generating and optimizing robot trajectories, achieving acceptable paint coverage in both simple and complex industrial settings.

## Method

Automatic robotic spray paint of Fanuc 250ib equipped with a titanium spray Bell end-effector was used for the trials. The industrial manipulator has six articulation axes. Each axis was servo-controlled using a resolver or feedback. The payload capacity of the end wrist was 15 kg and its reach was 2800 mm. The Fanuc 30ib controller controls the robot. The ISO 9001–ISO/TS 16,949 standard served as the foundation for experimentation.

Numerous factors, including paint flow, air shaping, turbine speed, high voltage, and viscosity influence robotic spray painting. Surface roughness together with thickness variation served as the primary output factors because they determine coating quality and functional performance along with operational life in industrial spray paint applications. The paint’s performance elements like material efficiency and corrosion resistance depend directly on its thickness measurement. Ununiform paint thickness leads to chipping, peeling and excessive paint buildup which damages both appearance and durability of paint coatings. The condition of surface roughness determines paint adherence and also controls product gloss and aerodynamic qualities particularly when used in automobile and aerospace industries. The primary factors influencing the performance were the pressure, distance, temperature, viscosity, humidity, and speed of the paint gun. Typically, handbook values or past experience are used to determine the levels of these parameters. After a week of continuous observation of the target process, relevant data were recorded, and the levels for each control parameter were determined, as shown in Table 1.

**Table 1:** Control factors and its levels.

Control Factors/Levels	A Viscosity (cps)	B Pressure (Bar)	C Distance (mm)	D Speed (mm/s)	E Temperature (°C)	F Humidity (%)
1	50	2	100	75	18	40
2	75	2.125	112.5	82.5	21.5	50
3	100	2.25	125	90	25	60

**Fig. 1:**
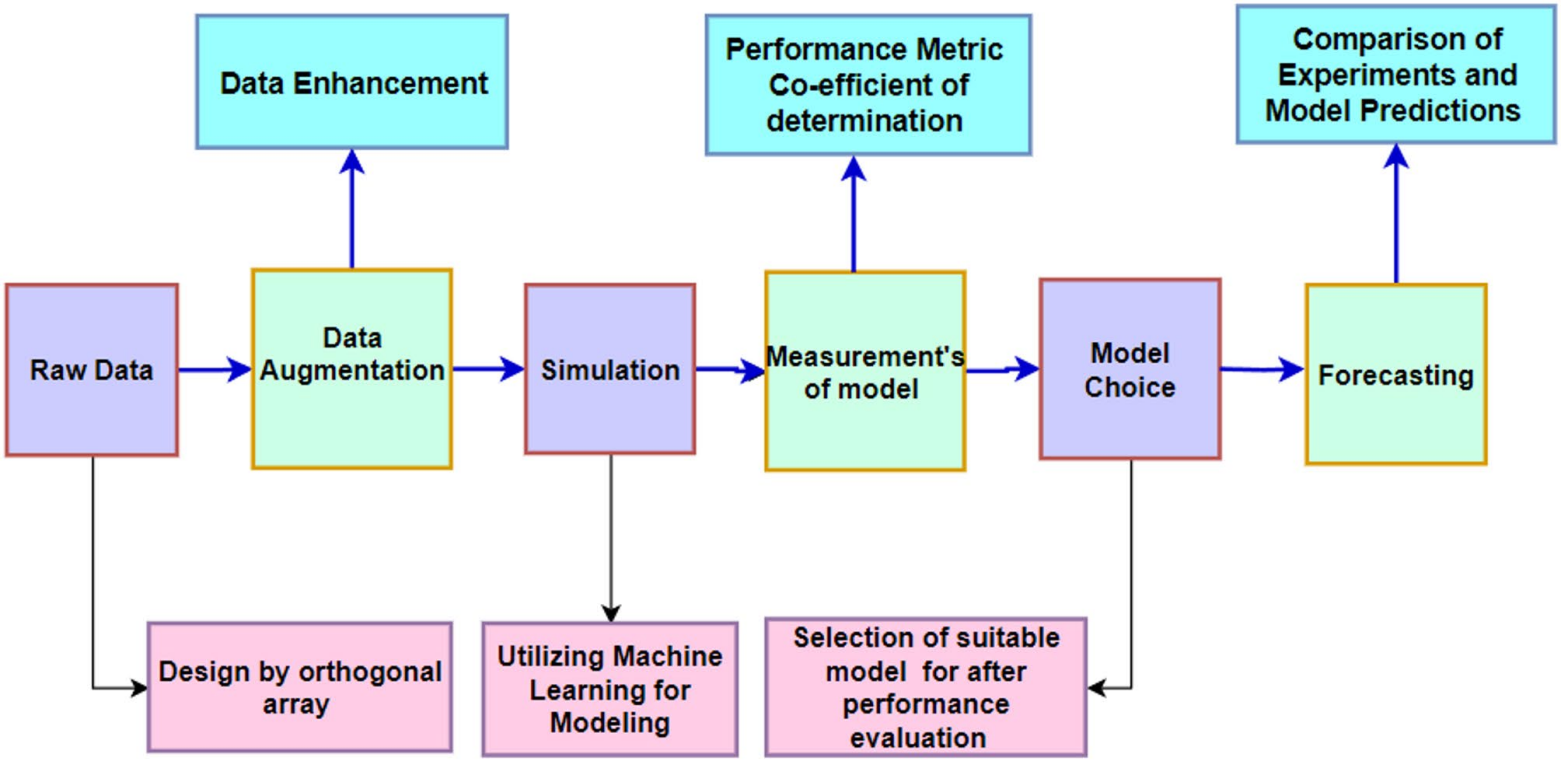
Data expansion and machine-learning analysis Layout.

Taguchi’s Design of Experiments is a powerful method used to analyze the impact of process control factors. Implementation of Taguchi Design of Experiments included an L27 orthogonal array because it works efficiently for optimizing six process parameters at two levels each. Testing all possible 2^6^ = 64 trials in a full factorial experiment proves too cumbersome because of time limits and resource availability. The L27 array enables experimental research through only 27 trials and effectively detects main elements of every factor. Results demonstrate that the L27 design approach from Taguchi offers precise parameter importance findings through a minimum number of experiments thus making it attractive for industrial process enhancements. The practice of three-level factor selection finds frequent use in first-stage optimization studies to detect important parameters prior to conducting thorough multi-level evaluations. Additionally, this method has been widely utilized by researchers to effectively plan experiments and draw objective conclusions. It also helps identify potential interactions between the selected process parameters. A detailed layout of the experimental data expansion and its analysis using machine learning are provided in Table 1; Fig. 1.

The framework combines Taguchi methodology with Random Forest implementation as an optimization toolkit. An L27 orthogonal array from the Taguchi method creates an organized experimental design which requires fewer tests while effectively recording the primary impacts of various input factors. Its application performs well in initial optimization phases because it requires substantially fewer experimental tests than exhaustive methods. After the experiment’s completion the S/N analysis of Taguchi method detects which input variables contribute most to response variability reduction.

Random Forest enhances predictive modeling precision by discovering hidden non-linear patterns in the data while it proceeds the analysis. The predictive model will generalize effectively to unobserved parameter combinations through the implementation of Random Forest following Taguchi trials. This dual methodology consolidates experimental design while enhancing the methodological depth and dependency of insights towards spray painting trajectory optimization.

### Modeling of spray painting

Although the parametric representation is computationally precise, its localized nature poses challenges for programmed path generation. However, the non-parametric triangular proximity of the surfaces of the parts was used to achieve this objective. In addition, when using a non-parametric representation, it is more convenient to establish connections between triangles compared to a parametric representation, where multiple surfaces need to be stitched together. Furthermore, the error that arises when converting a smooth surface into triangles can be minimized by reducing the size of the triangle. Triangular proximity (F) is used in this work to quickly create spray gun paths and make efficient use of the workspace of the robot.

1 $$F = \Delta _{m} :\left( {m = 1,2,....t} \right)$$

Where $\:{\Delta\:}_{m}$ denotes the mth triangle of the free-form surface’s m^th^ triangle and t represents the actual number of triangles. Triangles are characterized by three nodes, which can be represented by

2 $$\:{\Delta\:}_{m|ijk}={\Delta\:}_{m}({D}_{i},{D}_{j},{D}_{k}):(m=\text{1,2},\ldots t);$$

Where $\:{\Delta\:}_{m}$ consists of three nodes, and D_i_, D_j_, D_k_, and t represent the total number of nodes. The global coordinate scheme provides the x-, y-, and z-coordinates for each node.

3 $$\:{D}_{v|x,y,z}=\left\{{D}_{v}(x,y,z):v=\text{1,2},\ldots n\right\}$$

Because the location and surface geometry of the object to be coated are known in multidimensional Euclidean space with respect to a rigid reference frame, it is assumed that the object is immobile. Within the same reference frame, a six-dimensional vector function defines the spatial position and orientation of the spray gun.

4 $$\:{A}_{g}\left(t\right)={\left[{\rho\:}_{g}\left(t\right)*{\theta\:}_{g}\left(t\right)\right]}^{{\prime\:}}$$

Here, $\:{\rho\:}_{g}\left(t\right)$ is the position of the gun axis and $\:{\theta\:}_{g}\left(t\right)$ is the orientation, which are denoted in Eqs. (5) and (6).

5 $$\:{\rho\:}_{g}\left(t\right)={\left[{\rho\:}_{g}^{x}\left(t\right)*{\rho\:}_{g}^{y}\left(t\right)*{\rho\:}_{g}^{z}\left(t\right)\right]}^{{\prime\:}}$$

6 $$\:{\theta\:}_{g}\left(t\right)={\left[{\theta\:}_{g}^{x}\left(t\right)*{\theta\:}_{g}^{y}\left(t\right)*{\theta\:}_{g}^{z}\left(t\right)\right]}^{{\prime\:}}$$

**Fig. 2:**
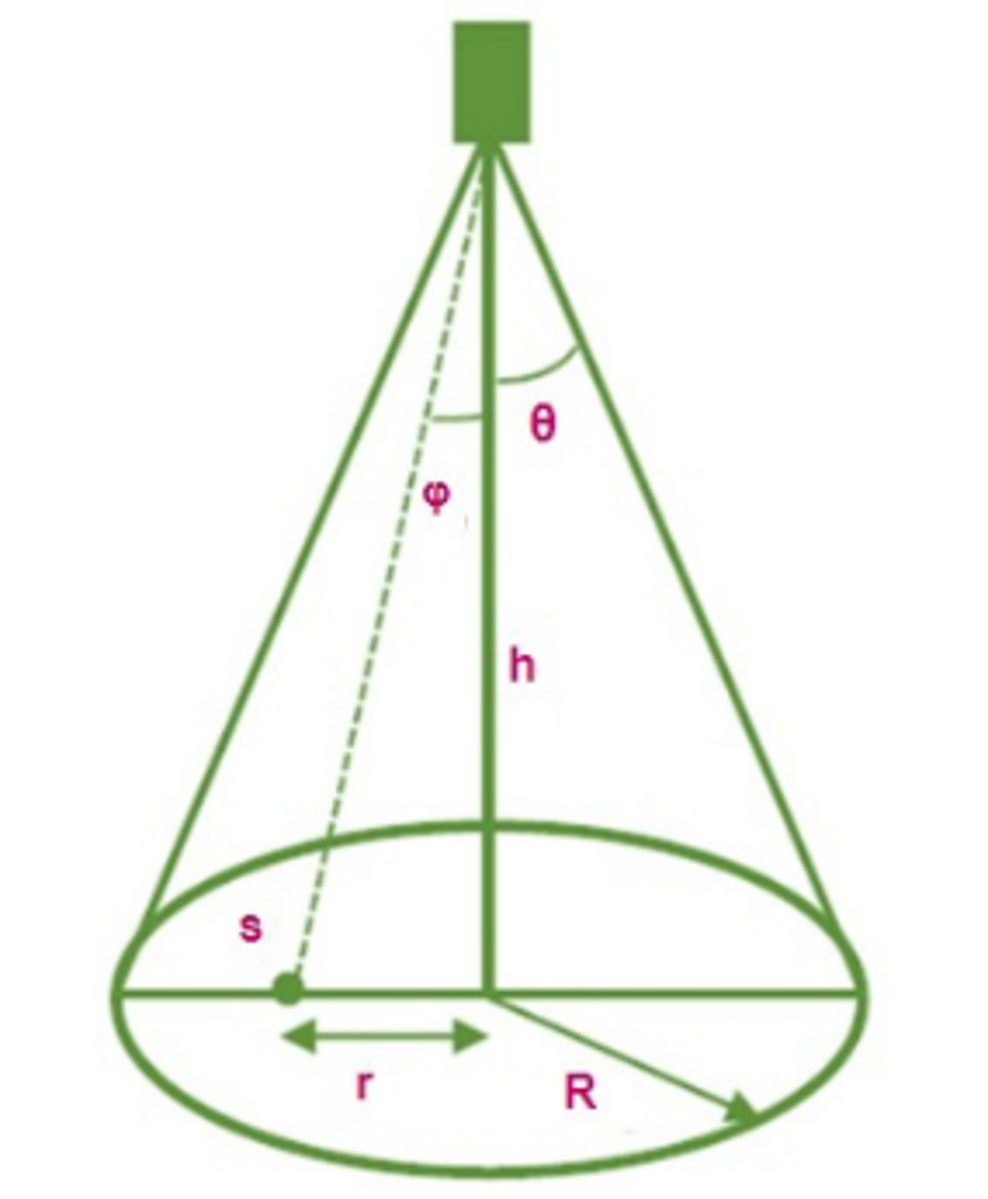
Model of the paint spray gun.

Whenever a gun is positioned in close proximity to a flat surface, it is hypothesized that the paint droplets are arranged in a conical shape, resulting in a roughly circular deposition pattern. The spray fan angle is represented by the symbol, ω. The spray gun standoff is denoted by d. t denotes the spray radius. T, on the other hand, represents the distance from any point S inside the spray circle. It is assumed that the material’s deposition rate remains constant. Figure 2 represents the model of the spray gun used in the proposed work.

It is necessary to understand the deposition rate or flux to create the spray gun trajectories. The flux at the surface point is dependent on the outlines of the painted surface as well as the location and orientation of the spray gun. Researchers often simulate paint distributions using Parabolic, Gaussian, and Beta distributions. The paint dispersion in this model is related to the distance to the tool center. This model was largely approximated and caused considerable variations in the surface spray paint thickness. Equation for paint flux $\:\dot{\phi\:}\left(T\right)\:$from spray circle center

7 $$\dot{\varphi} \left(T\right)=\frac{2{\gamma\:}_{o}}{\pi\:{t}^{2}}\left(1-\frac{{T}^{2}}{{t}^{2}}\right),\:-t\le\:T\le\:t,\:t=d*tan\alpha\:$$

$\:{\gamma\:}_{o}\:$represents maximum flow rate, and α represents spray fan angle.

The following equation gives the paint flux,

8 $$\:{a}_{g}^{t}(x,y)={a}_{g,max}^{t}*exp\left(-\frac{1}{2}\left[{\left(\frac{x}{{\tau\:}_{x}}\right)}^{2}+{\left(\frac{y}{{\tau\:}_{y}}\right)}^{2}\right]\right)$$

Where, $\:{a}_{g,max}^{t}\:$is the maximum coating value, and $\:{\tau\:}_{x}$ and $\:{\tau\:}_{y}$ represent the Gaussian parameters over the x- and y-axes, respectively.

This study utilized a beta distribution model. This model can be chosen. In addition, the model yielded various distributions.

9 $$\dot{\varphi } \left(T\right)=\gamma\:\:{\left(1-\frac{{T}^{2}}{{t}^{2}}\right)}^{\beta\:-1},\:for\:-t\le\:T\le\:t,\:t=d*tan\alpha\:$$

The film accumulation can be determined at an arbitrary surface point. The flux, , is dependent on β, t, transfer efficiency $\:\eta\:$, and flow rate $\:{\gamma\:}_{o}$. It can be calculated using the Eq. (10).

10 $$\dot{\varphi } \left(T\right)=\frac{\eta\:{\gamma\:}_{o}\beta\:}{\pi\:{t}^{2}}\:{\left(1-\frac{{T}^{2}}{{t}^{2}}\right)}^{\beta\:-1},\:for\:-t\le\:T\le\:t,\:t=d*tan\alpha\:$$

The paint was sprayed onto the surface while the spray gun moved relative to it. To make the most of the paint dispersion model and compute the travel time, the authors of this study assumed a constant spray gun velocity. Professionals in automobile spray painting lend support to this point. The following formula can be used to determine the thickness of the paint at various locations.

11 $$\:\phi\:\left(T\right)=\underset{0}{\overset{t}{\int\:}}\dot{\phi\:}\left(T\right)dt,\:\:-t\le\:T\le\:t\:$$

12 $$\:\phi\:\left(T\right)=\underset{0}{\overset{t}{\int\:}}\frac{\eta\:{\nu\:}_{o}\beta\:}{\pi\:{t}^{2}}{\left(1-\frac{{T}^{2}}{{t}^{2}}\right)}^{\beta\:-1}.dt\:for\:\:-t\le\:T\le\:t\:$$

Assuming linearity,

13 $$\:x=2\sqrt{{t}^{2}-{T}^{2}}$$

14 $$\:t=\frac{x}{\mu\:}=\frac{2\sqrt{{t}^{2}-{T}^{2}}}{\mu\:}$$

The thickness of the paint during the paint gun traveling along the X-axis can be determined using the following formula:

15 $$\phi\:\left(T\right)=\:\frac{4\eta\:{\nu\:}_{o}\beta\:}{\pi\:{\mu\:t}^{2\beta\:}}\left(\frac{{\left({t}^{2}-{T}^{2}\right)}^{\beta\:-0.5}}{2\beta\:-1}\right)\:for\:\:-t\le\:T\le\:t$$

Spraying requires control of the gun stand-off distance, spray gun velocity, and paint overlap to achieve the desired paint thickness. This study determined the optimum paint overlap for two paint passes. $\:{\phi\:}_{1}\left(T\right)$ and $\:{\phi\:}_{2}\left(T\right),\:$are the paint deposits of paints 1 and 2, respectively. The paint thickness model is given in Eqs. (16)–(20).

16 $$\:f(T,h)=\left\{\begin{array}{c}{\phi\:}_{1}\left(T\right),\:-t\le\:T\le\:t-h\\\:{\varphi\:}_{1}\left(T\right)+{\phi\:}_{2}\left(T\right),\:t-h\le\:T\le\:t\\\:{\phi\:}_{2}\left(T\right),\:t\le\:T\le\:3t-h\end{array}\right.$$

17 $$\:{\phi\:}_{1}\left(T\right)=\underset{0}{\overset{{t}_{1}}{\int\:}}f\left({T}_{1}\right).dt,\:-t\le\:{T}_{1}\le\:t$$

18 $$\:{\phi\:}_{2}\left(T\right)=\underset{0}{\overset{{t}_{2}}{\int\:}}f\left({T}_{2}\right).dt,\:t-h\le\:{T}_{2}\le\:3t-h$$

19 $$\:{t}_{2}=\frac{\sqrt{{t}^{2}-{\left(2t-h-T\right)}^{2}}}{\mu\:}$$

To determine the ideal overlap distance over a specified paint thickness, qd, the overlap thickness fluctuation was minimized.

For 0 < T ≤ 2t − h, f(T, h) has maximum and minimum thickness values. The objective function E is unaffected by changes in the spray gun velocity, as shown.

21 $$\:\underset{{h\epsilon\:}\left[0,t\right]}{\text{min}}E={\left({f}_{maximum}-{f}_{minimum}\right)}^{2}$$

Calculated $\:{\mu\:}_{optimum}\:$using the h range of 0 to t. For every h in the range, $\:{\mu\:}_{optimum}$ is calculated first and replaced in f(T, h) to calculate E throughout the iterations.

22 $$\mu _{{optimum}} = \frac{{\left( {1/2t - h} \right)\int\nolimits_{{2t - D}}^{0} {W^{2} \left( {T,h} \right).dT + W_{{max}}^{2} \left( h \right) + W_{{min}}^{2} \left( h \right)} }}{{\phi _{h} \left[ {\left( {1/2t - h} \right)\mathop \int\nolimits_{{2t - D}}^{0} W^{2} \left( {T,h} \right).dT + W_{{max}}^{2} \left( h \right) + W_{{min}}^{2} \left( h \right)} \right]}}$$

## Taguchi’s design of experiments

In this experimental layout, we adopted the S/N ratio characteristics to evaluate porosity, surface roughness, and hardness. The goal was to minimize the porosity and surface roughness while maximizing hardness. These equations provided the necessary guidelines for this evaluation.

23 $$\:{\text{S/N}}_{\text{LtB}}\text{=-10log}\left[\frac{\text{1}}{{ \omega }}\sum\limits_{\text{i=1}}^{{ \omega }}\frac{\text{1}}{{\text{Z}}_{\text{i}}^{\text{2}}}\right]$$

Typically, three scenarios of quality characteristics are used to determine the signal-to-noise (S/N) ratio: Larger-the-Better (LtB), Nominal-the-Better (NtB), and Smaller-the-Better (StB). This study examines the relationship between Thickness Variation and Surface Roughness with the objective of minimizing these factors. The formula for calculating the signal-to-noise (S/N) ratio is expressed mathematically.

24 $$\:{\text{S/N}}_{\text{NtB}}\text{=-10log}\left[\frac{\text{1}}{ \omega }\sum\limits_{\text{i=1}}^{ \omega }{\left({\text{z}}_{\text{i}}\text{-}{\text{z}}_{\text{0}}\right)}^{\text{2}}\right]$$

ω indicates the total number of experiments, and Z_i_ denotes the reaction in the i_th_ experiment.

25 $$\:{\text{S/N}}_{\text{StB}}\text{=-10log}\left[\frac{\text{1}}{ \omega }\sum\:_{\text{i=1}}^{ \omega }{\text{Z}}_{\text{i}}^{\text{2}}\right]$$

## Random forest regression

**Fig. 3:**
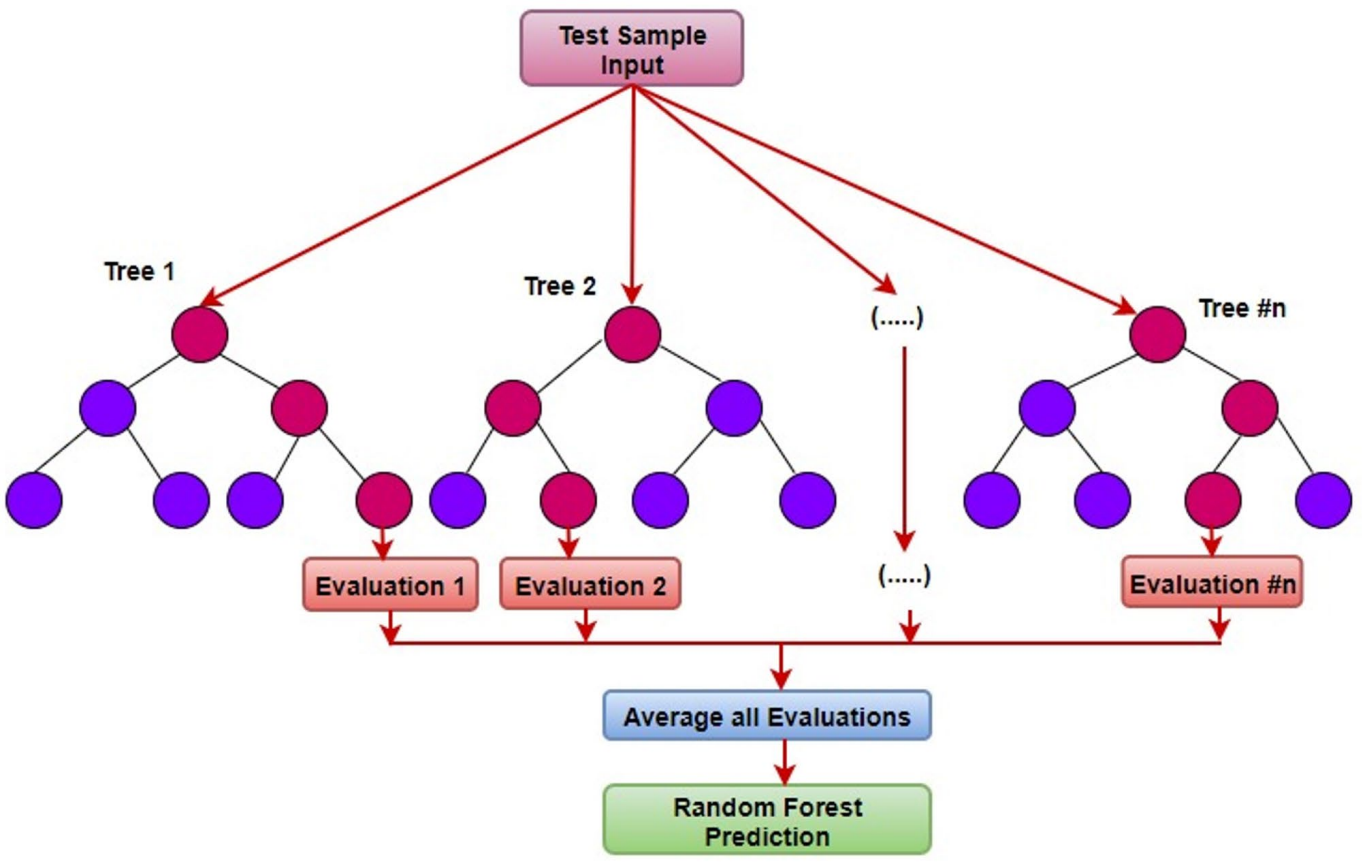
Schematic outline of algorithm.

Regression analysis is a statistical approach used to create a model of the relationship between the input data and target variable. This study used the Random Forest (RF) method to carry out the regression for this investigation. Random Forest regression proved effective because it manages nonlinear interactions with ease during spray painting operations. The model achieved its configuration using 100 decision trees that reached heights up to 10 levels while selecting features according to the square root of the total features to enhance both predictive power and runtime speed. The Random Forest model showed superior performance than traditional regression approaches when calculating paint thickness variation and surface roughness which made it an effective tool for optimization tasks. This ensemble machine-learning approach builds several decision trees during the training phase. Then, during the testing phase, it averaged the collective predictions from all decision trees. Figure 3 shows a schematic outline of the random forest regression.

Here is a summary and description of the proposed procedure:

Step1: **Start**.

Commence the process.

Step 2: **Define Target Parameters**.

The goal structural parameters of the spray paint should be established and then stated.

Step 3: **Set Parameter Bounds**.

It is also important to determine the lower and upper limits of each target parameter.

$$L_{o}^{b} = \left[ {L_{{O1}} ,L_{{O2}} ,....L_{{OM}} } \right]$$

$$U_{o}^{b} = \left[ {U_{{O1}} ,U_{{O2}} ,....U_{{OM}} } \right]$$

Here, $\:{L}_{o}^{b}$ and $\:{U}_{o}^{b}$ represents the bounds (upper and lower).

Step 4: **Sampling Phase**.

Derive or compute Z vectors by randomly selecting values within the prescriptive dimensional limits that are assigned to them.

$$Y_{z} = L^{b} + \left( {U^{b} - L^{b} } \right)~o~r_{Z} ~for~z = 1,2,...Z$$

Step 5: **Compute Structural Responses**.

Find the values of the structural response X_z_ for each sample z, and compile the results.

$$\:{X}_{z}=f\left({y}_{Z}\right)$$

Step 6: **Form Matrices**.

Gather all X_z_ vectors and store them in the attribute matrix x.

$$\:x=\left[\begin{array}{c}{X}_{z1}\\\:{X}_{z2}\\\:{X}_{zn}\end{array}\right]$$

Gather all Y_z_ vectors and store them in the attribute matrix y.

$$\:y=\left[\begin{array}{c}{Y}_{z1}\\\:{Y}_{z2}\\\:{Y}_{zn}\end{array}\right]$$

Step 7: **Train Regression Model**.

Employ the vectors of input value matrices x and y in the training of $\:\widehat{h}$ which is the regression model.

$$\:\widehat{h}\left(x\right)\approx\:y$$

Step 8: **Model Updating Phase**.

A new set of responses is obtained in terms of the experimental structure. Employ the results obtained from the regression model to determine the forecast of new target characteristics $\:\widehat{Y}$.

$$\:\widehat{Y}=\widehat{h}\left(\widehat{X}\right)$$

Step 9: **Update Model**.

- Incorporate the predicted parameters $\:\widehat{Y}$ into the model.

Step 10: **End**.

Each decision tree is constructed using a process known as “bootstrap aggregation,” given a complete set of training data consisting of Z. Z samples for both input and output variables. This process involves random selection with replacement, which is a subset of the available input samples. Consequently, many decision trees may include a single sample. The process of creating a tree begins with a root node and its seed input sample. For the Gini Index-based “entropy” to go down, a “splitting criterion” divides the root node into two child nodes. Splitting factors in “feature subsampling” include assigning a feature a cutoff from a group of features. “Leaf nodes” show the decision tree’s end forecasts after hitting a “stopping criterion” by the iterative process.

It is common to create a leaf node when the amount of data drops below a certain level, which is usually five for regression work. Training of the random forest is accomplished through repeated use of the decision tree process. During testing, the decision trees of the random forests look at new input samples and produce outputs. This results in the forecast of the overall tree, after arriving at the sum of the tree predictions, and then dividing by three.

Random forests offer a fairly accurate assessment of unknown data success. Creating a decision tree without of bag or OOB samples which are the samples which were not used in bootstrapping. There is a way through which the random forest model comes up with its results, and this is by summing the mean squared error of every decision tree that has made the accuracies. Owing to the multiple decision tree average, it can easily deal with factors that are missing from input data, reduce feature normalization, and handle errors better than other machine learning regression methods.


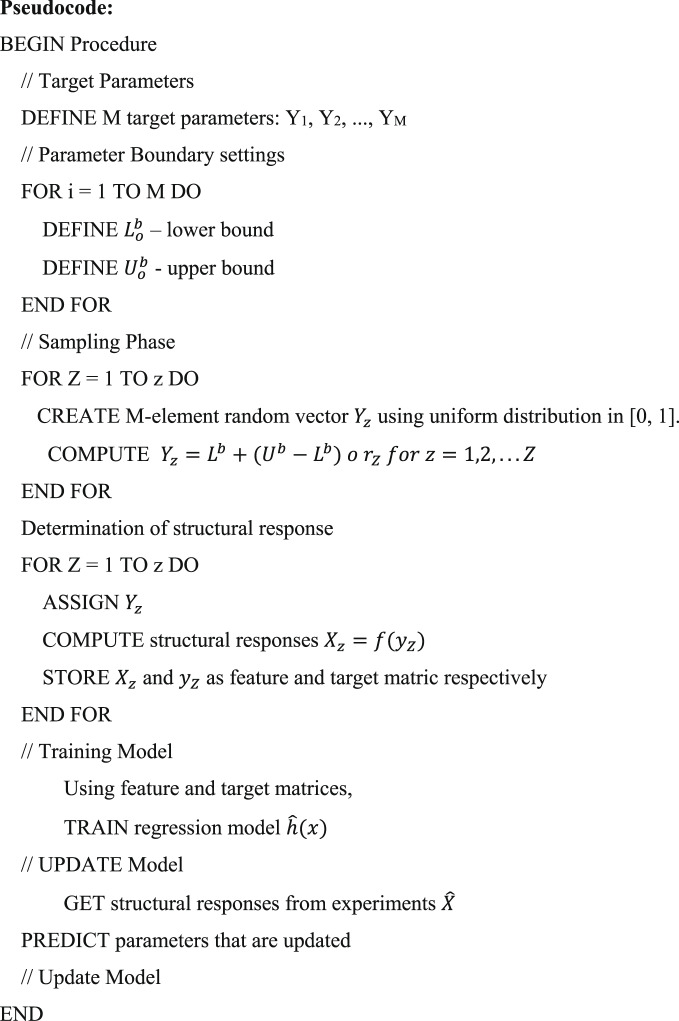


## Results and discussion

The tests were conducted on automated spray-painting using Taguchi’s orthogonal array DoE. The results of these experiments are presented in Table 2.

**Table 2:** Experimental results for the experiments conducted.

Trial No.	Performance Characteristics							
	A	B	C	D	E	F	Thickness Variation (µm)	Surface Roughness (µm)
1	1	1	1	1	1	1	2.289	9.39
2	1	1	1	1	2	2	2.159	21.28
3	1	1	1	1	3	3	2.639	23.63
4	1	2	2	2	1	1	2.737	25.58
5	1	2	2	2	2	2	2.797	26.75
6	1	2	2	2	3	3	2.986	28.75
7	1	3	3	3	1	1	2.658	30.67
8	1	3	3	3	2	2	2.573	31.68
9	1	3	3	3	3	3	3.005	34.81
10	2	1	2	3	1	2	2.619	32.83
11	2	1	2	3	2	3	2.749	41.29
12	2	1	2	3	3	1	2.952	15.71
13	2	2	3	1	1	2	3.087	34.18
14	2	2	3	1	2	3	3.164	35.68
15	2	2	3	1	3	1	3.433	26.53
16	2	3	1	2	1	2	2.662	1.98
17	2	3	1	2	2	3	2.952	12.86
18	2	3	1	2	3	1	3.29	2.11
19	3	1	3	2	1	3	2.136	3.64
20	3	1	3	2	2	1	2.169	1.08
21	3	1	3	2	3	2	2.215	2.09
22	3	2	1	3	1	3	2.307	18.88
23	3	2	1	3	2	1	2.339	22.63
24	3	2	1	3	3	2	2.588	13.74
25	3	3	2	1	1	3	2.605	22.76
26	3	3	2	1	2	1	2.875	21.73
27	3	3	2	1	3	2	2.957	12.26

Effect on Thickness Variation and Surface Roughness. Using Eq. (24), the S/N of the Thickness Variation and Surface Roughness of the responses were estimated, and the S/N values obtained. The computations of S/N ratio occurred for thickness variation and surface roughness within the 27 experimental trials that implemented Taguchi’s DoE approach as shown in Table 3. This study seeks to minimize surface roughness and thickness variation, so the “Smaller-the-Better” quality characteristic became the basis to calculate S/N ratios. The ratio’s high value shows an improved operational capability due to lower uncertainty levels. The measurement results from Experiments demonstrated the higher value of S/N ratio for thickness variation indicating the optimal parameters for minimizing thickness variation. Experiments showed the highest S/N ratio for surface roughness which indicates that its associated parameter combinations yield optimal results for reducing surface roughness. The table identifies optimal control parameters for achieving high-quality consistent spray coatings while minimizing deviations in target performance characteristics.

**Table 3:** S/N ratio values of thickness variation and surface roughness.

Trial No.	Thickness Variation (dB)	Surface Roughness (dB)
1	− 7.19	− 19.46
2	− 6.69	− 26.56
3	− 8.43	− 27.46
4	− 8.75	− 28.16
5	− 8.93	− 28.55
6	− 9.5	− 29.17
7	− 8.49	− 29.73
8	− 8.21	− 30.02
9	− 9.56	− 30.84
10	− 8.36	− 30.33
11	− 8.79	− 32.31
12	− 9.4	− 23.93
13	− 9.79	− 30.68
14	− 10	− 31.05
15	− 10.71	− 28.47
16	− 8.5	− 5.94
17	− 9.4	− 22.19
18	− 10.34	− 6.49
19	− 6.59	− 11.22
20	− 6.73	− 0.67
21	− 6.91	− 6.41
22	− 7.26	− 25.52
23	− 7.38	− 27.09
24	− 8.26	− 22.76
25	− 8.32	− 27.15
26	− 9.17	− 26.74
27	− 9.42	− 21.77

A detailed response table of the mean S/N ratio for thickness variation and surface roughness is shown in Tables 4 and 5, respectively.

**Table 4:** Response table for thickness variation.

Level	A	B	C	D	E	F
1	− 18.45	− 17.63	− 18.16	− 18.86	− 18.16	− 18.67
2	− 19.50	− 18.97	− 19.04	− 18.39	− 18.37	− 18.36
3	− 17.75	− 19.11	− 18.51	− 18.46	− 19.18	− 18.67
Delta	1.75	1.47	0.88	0.47	1.03	0.31
Rank	1	2	4	5	3	6

The response table present in Table 4 displays how thickness variation responds to control factors A through F while operating at varying levels. The Delta value from each factor shows its effectiveness by measuring their S/N ratio extremes. The strength of thickness variation depends on the Delta value. The highest delta value of 1.75 belongs to viscosity (A) establishing it as the leading factor that drives thickness variation results. The data demonstrates that controlling paint viscosity results in superior and uniform paint thickness results. Among the influencing factors Temperature (E) and pressure (B) stand as the second most important elements following viscosity (A). The testing of humidity (F) showed the lowest Delta value which indicates its minimal role in thickness changes compared to the other control conditions. The achievement of uniform thickness in robotic spray painting requires the exact management of viscosity and following control of both temperature and pressure.

**Table 5:** Response table for surface roughness.

Level	A	B	C	D	E	F
1	− 28.80	− 22.68	− 25.07	− 28.41	− 26.32	− 23.43
2	− 26.08	− 28.89	− 28.75	− 19.97	− 25.31	− 25.78
3	− 22.40	− 25.71	− 23.46	− 28.90	− 25.64	− 28.07
Delta	6.41	6.21	5.30	8.94	1.01	4.64
Rank	2	3	4	1	6	5

The response table in Table 5 depicts mean S/N ratio changes per control factor level for surface roughness analysis. The Delta values in this table indicate the strength of impact each variable has on the final surface roughness results. The study findings demonstrated that D-speed exhibited the largest change in Delta value at 8.94 thus establishing itself as the prime factor for controlling surface roughness. The control of spray speed serves as a vital operational factor when trying to create smooth surfaces in robotic spray-painting operations. The measurements of viscosity (A) and pressure (B) contributed to surface roughness variation yet remained lower than speed. Temperature (E) and humidity (F) show less impact on surface roughness than speed because speed plays the leading role in shaping surface finish. The obtained findings demonstrate that controlling spray speed remains critical to achieve optimal surface finish with minimum roughness but technical adjustments between parameters will enhance surface refinement ability.

**Fig. 4:**
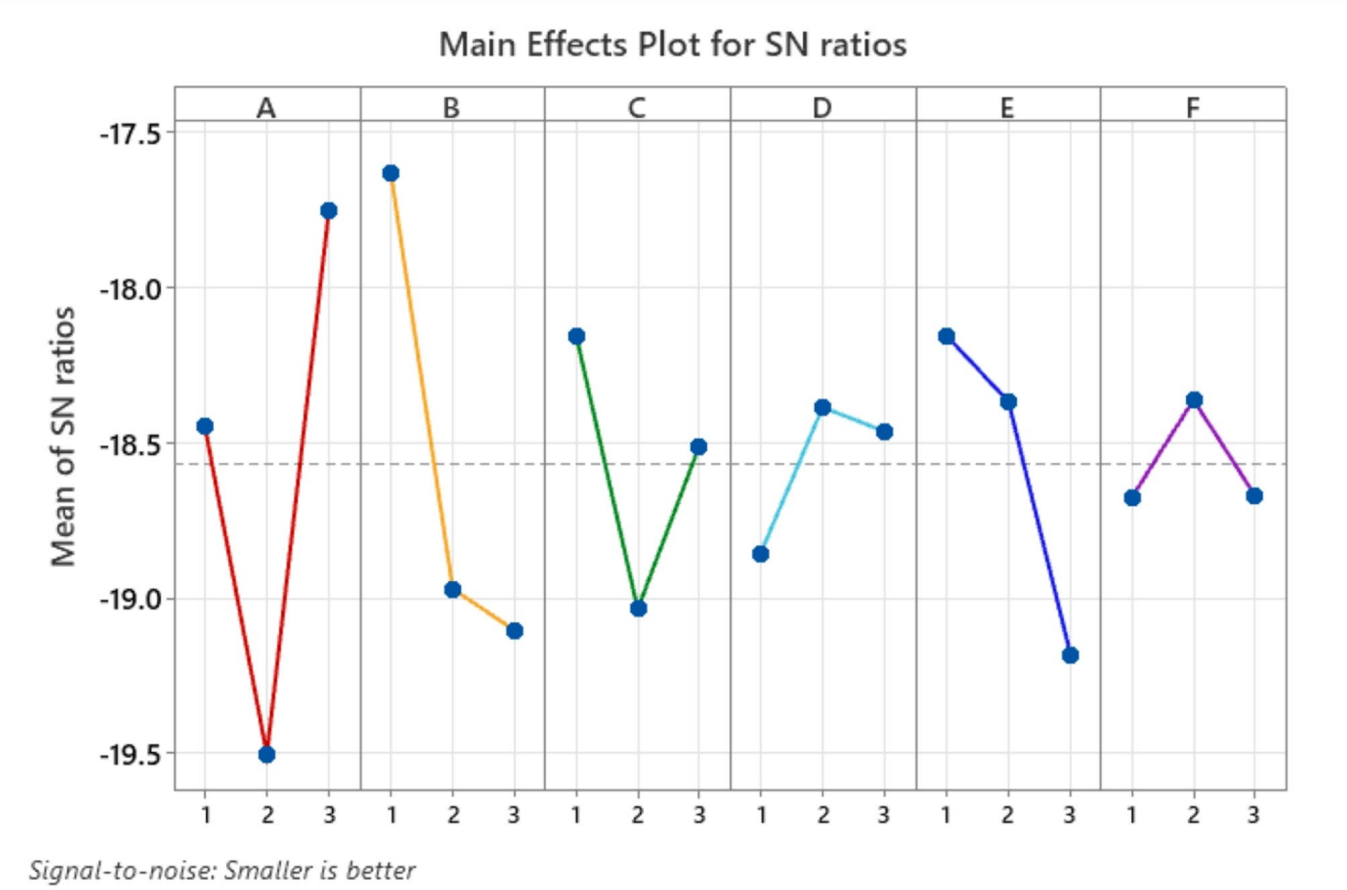
Effect of Control Factors on Thickness Variation.

The S/N ratio analysis of robotic spray-painting thickness variation depends on control factors which include viscosity, pressure, distance, speed, temperature, and humidity as shown in Fig. 4. The S/N ratio enhances steadily throughout the tested spray distances from 100 mm to 125 mm since better gun-to-surface separation creates homogenous coating thickness. The S/N ratio decreases slightly when pressure increases from 2 to 2.25 bar thus indicating pressure serves as a limited influencing factor for process variation. Increasing the temperature from 18 °C to 25 °C creates a positive effect on thickness uniformity as it results in a considerable rise of S/N ratio. The study proves that elevated temperatures create better paint spreading conditions which leads to improved thickness control. When humidity rises from 40 to 60% the S/N ratio decreases fast which shows that environmental moisture causes stability problems in spray applications leading to formation of irregularities. The S/N ratio shows a mild enhancement due to increases in speeds when the range goes from 75 mm/s to 90 mm/s because speed affects thickness consistency in a moderate fashion. The S/N ratio experiences a significant drop when viscosity ranges from 50 cps to 100 cps as this elevation of paint thickness significantly affects sample variation when measured. Figure 4 demonstrates that minimal thickness variation requires increase of spray distances combined with high temperatures and low viscosity and humidity values.

**Fig. 5:**
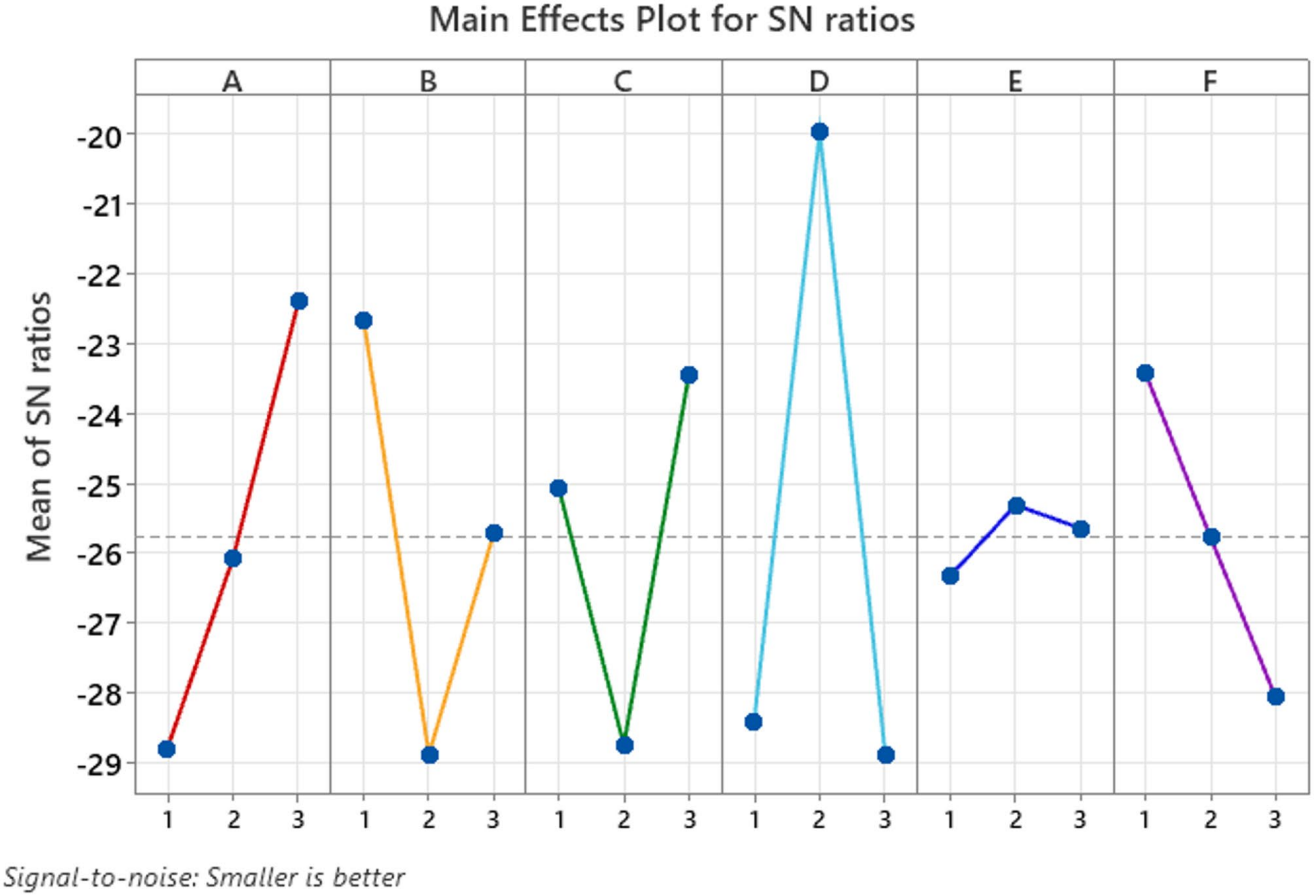
Effect of Control Factors on Surface Roughness.

Figure 5 illustrates how surface roughness S/N ratio changes when using the control factors viscosity, pressure, distance, speed, temperature, and humidity in robotic spray painting. An increase of spray distance from 100 mm to 125 mm leads to increased S/N ratio which indicates that extended distances produce surfaces with decreased roughness. The control variable pressure creates a small but insignificant positive impact on the analysis since S/N ratio results slightly better between 2 bar to 2.25 bar operating pressure. The surface smoothness suffers substantially when temperature rises from 18 °C to 25 °C because increasing the temperature leads to reduced S/N ratio while potentially causing irregularities due to quick drying. The S/N ratio shows a minor enhancement from 40 to 60% humidity levels because this controlled level of moisture creates positive effects on surface finish quality. The S/N ratio increases notably when spray speed reaches 90 mm/s while speed operates at 75 mm/s which generates a superior surface refinement. The S/N ratio rapidly declines when increasing viscosity from 50 cps to 100 cps since this viscosity level impedes paint flow and produces uneven surfaces. Figure 5 establishes that quality paint finishes can be achieved through maintaining higher spray distances along with fast speeds and keeping viscosity low along with temperature control.

A polynomial quadratic model, also referred to as a regression model, provides a description of the system behavior. Regression models were generated by performing a linear logarithmic transformation. The coefficients of the regression model were determined using Design Expert software version 11.0 in an empirical form. Analysis of variance was used to assess the significance of the parameters.

**Table 6:** ANOVA for thickness variation.

Source	DF	Adj SS	Adj MS	F-Value	*P*-Value
A	2	13.1918	6.59589	90.61	0.00000001
B	2	10.5367	5.26836	72.37	0.00000004
C	2	2.8722	1.43611	19.73	0.00008450
D	2	1.2092	0.60460	8.31	0.00418525
E	2	5.2813	2.64063	36.28	0.00000290
F	2	0.6434	0.32171	4.42	0.03252020
Error	14	1.0191	0.07279		
Total	26	34.7537			
R-sq-97.07%		R-sq(adj)- 94.55%		R-sq(pred)-89.09%	

The robotic spray-painting thickness variation data gets analyzed through Analysis of Variance (ANOVA) tests in Table 6. ANOVA reveals statistical importance and impact strength levels of viscosity (A) and pressure (B) and distance (C), speed (D) and temperature (E) and humidity (F) toward thickness variation. The analysis of variance results includes an examination of degrees of freedom (DF) as well as the adjusted sums of squares (Adj SS) and the adjusted mean squares (Adj MS) and F-values and p-values for every factor. An F-value quantifies the ratio between factor-explained variance and unexplained variance in the analysis and it demonstrates the importance of that factor. The p-value calculation determines statistical probability that a specific effect would happen randomly thus a low p-value below 0.05 signals significant influence from that factor. The data shows viscosity (A), and pressure (B) possess extremely high F-values (90.61 and 72.37, respectively) paired with p-values that fall below 0.05 indicating the highest statistical importance for altering thickness levels. The factors distance (C) and speed (D) and temperature (E) demonstrate significant relationships but exhibit lower F-values than viscosity (A) and pressure (B), while F-value for humidity (F) stands at 4.42 to reach statistical significance with a marginally sub-0.05 p-value. The statistical model achieves robustness due to its 97.07% R-squared value and 94.55% adjusted R-squared value which demonstrates that it effectively explains most changes observed in thickness variation.

**Table 7:** ANOVA for surface roughness.

Source	DF	Adj SS	Adj MS	F-Value	*P*-Value
A	2	361.31	180.66	15.37	0.00029358
B	2	311.45	155.72	13.25	0.00058969
C	2	252.79	126.39	10.75	0.00148069
D	2	859.88	429.94	36.58	0.00000276
E	2	43.87	21.94	1.87	0.19116462
F	2	127.07	63.54	5.41	0.01820431
Error	14	164.53	11.75		
Total	26	2120.91			
R-sq-92.24%		R-sq(adj)- 85.59%		R-sq(pred)-81.15%	

ANOVA results for surface roughness in robotic spray-painting process appear in Table 7. The ANOVA analysis establishes which control factors have the most significant impact on surface roughness measurements in the same manner it did for thickness measurements. Table 7 presents information about the degrees of freedom (DF) in addition to the adjusted sums of squares (Adj SS) and adjusted mean squares (Adj MS) and F-values and p-values for each control factor. Speed (D) stands out among all tested parameters because it demonstrates the highest F-value (36.58) along with the lowest p-value thus making it the primary deciding factor for surface roughness control. Viscosity (A), pressure (B), and distance (C) also show statistically significant effects with high F-values and p-values well below 0.05. The p-value of Temperature (E) exceeds 0.05 which indicates that this factor does not influence surface roughness at the examined level. The F-value of 5.41 and low p-value prove the moderate significance of Humidity (F). The selected control factors display an excellent model fit because the overall R-squared value measures 92.24% while the adjusted R-squared value reaches 85.59%. This indicates the model correctly captures most surface roughness variability. The findings validate the need to optimize all three variables of spray speed alongside viscosity and pressure to reach optimal smoothness of robotic spray painted surfaces.

After analyzing the ANOVA results from Tables 6 and 7 for the thickness variation and surface roughness, respectively, the most significant parameters were identified. These parameters are then included in the final computational representation of the relationships. The mathematical model relationships obtained are listed in Eqs. (26) and (27).

26 $$\begin{aligned} & {\text{Thickness}}\,{\text{Variation}} = {\text{8}}.{\text{5585 }} - 0.{\text{1419 A}}^{{\text{1}}} + 0.{\text{9181 A}}^{{\text{2}}} - 0.{\text{7763 A}}^{{\text{3}}} - 0.{\text{8819 B}}^{{\text{1}}} + 0.{\text{3948}} \\ & {\text{B}}^{{\text{2}}} + 0.{\text{487}}0{\text{ B}}^{{\text{3}}} - 0.{\text{3974 C}}^{{\text{1}}} + 0.{\text{4}}0{\text{15 C}}^{{\text{2}}} - 0.00{\text{41 C}}^{{\text{3}}} + 0.{\text{2993 D}}^{{\text{1}}} - 0.{\text{153}}0{\text{ D}}^{{\text{2}}} - 0.{\text{1463 D}}^{{\text{3}}} \\ & \quad - 0.{\text{4196 E}}^{{\text{1}}} - 0.{\text{1919 E}}^{{\text{2}}} + 0.{\text{6115 E}}^{{\text{3}}} + 0.{\text{1259 F}}^{{\text{1}}} - 0.{\text{2174 F}}^{{\text{2}}} + 0.0{\text{915 F}}^{{\text{3}}} \\ \end{aligned}$$

27 $$\begin{aligned} & {\text{Surface}}\,{\text{Roughness }} = {\text{ 23}}.{\text{358 }} + {\text{ 4}}.{\text{414 A}}^{{\text{1}}} + 0.{\text{13}}0{\text{ A}}^{{\text{2}}} - {\text{ 4}}.{\text{544 A}}^{{\text{3}}} - {\text{ 3}}.{\text{541 B}}^{{\text{1}}} + {\text{ 4}}.{\text{581 B}}^{{\text{2}}} \\ & \quad - {\text{1}}.0{\text{39 B}}^{{\text{3}}} - {\text{ 2}}.{\text{973 C}}^{{\text{1}}} + {\text{ 4}}.{\text{21}}0{\text{ C}}^{{\text{2}}} - {\text{ 1}}.{\text{237 C}}^{{\text{3}}} + {\text{ 3}}.{\text{235 D}}^{{\text{1}}} - {\text{ 7}}.{\text{936 D}}^{{\text{2}}} + {\text{ 4}}.{\text{7}}0{\text{1 D}}^{{\text{3}}} - 0.{\text{226 E}}^{{\text{1}}} \\ & \quad + {\text{1}}.{\text{662 E}}^{{\text{2}}} - {\text{ 1}}.{\text{436 E}}^{{\text{3}}} - {\text{ 2}}.{\text{165 F}}^{{\text{1}}} - 0.{\text{8}}00{\text{ F}}^{{\text{2}}} + {\text{ 2}}.{\text{965 F}}^{{\text{3}}} \\ \end{aligned}$$

The most crucial method for determining whether a regression model is a good fit is to graphically analyze the residuals. The amount by which a regression line misses a data point vertically is expressed as a residual value. The regression lines provided the best fit for a set of data.

**Fig. 6:**
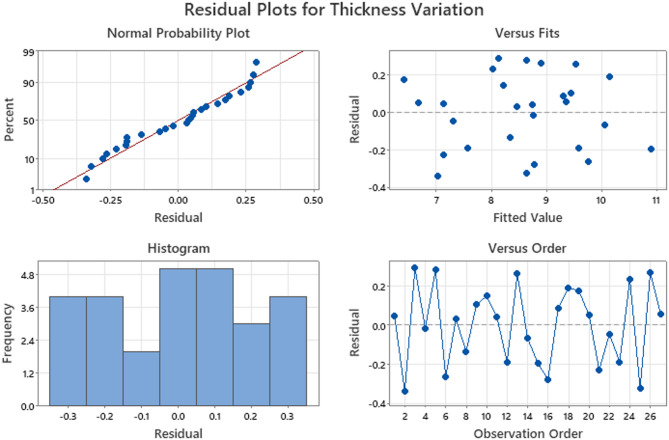
Residual Plots of Thickness Variation.

**Fig. 7:**
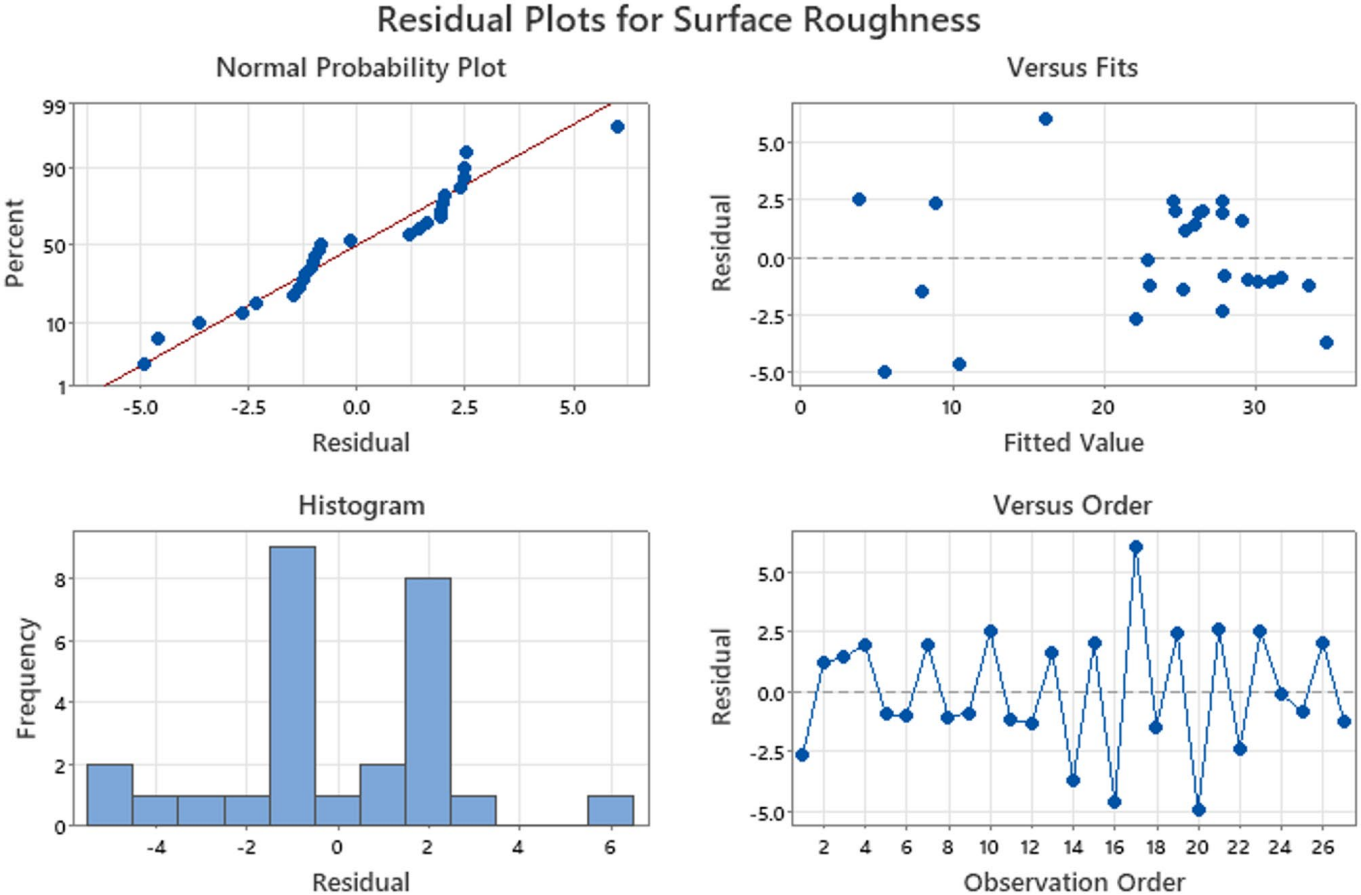
Residual Plots of Surface Roughness.

The correlation between the anticipated values and trial run experiments is shown in the residual plots. Residual plots of the thickness variation and surface roughness parameters are shown in Figs. 6 and 7, respectively. As seen in the probability plot, the points were distributed linearly within a range. This indicates that the trial run (experiment) and expected values have a stronger association.

In the versus fit, the residual and predicted values are contrasted. There appears to be very little difference between them because they are both comparatively close to one another, at approximately. Histogram plots show unambiguous data regarding the residuals. Finally, an analysis of the residual versus the observations was performed. The residual values were observed to be within the positive and negative ranges respectively, suggesting the existence of specific correlations. The models show promise for adequacy based on a thorough investigation of thickness variation and surface roughness residual plots.

**Fig. 8:**
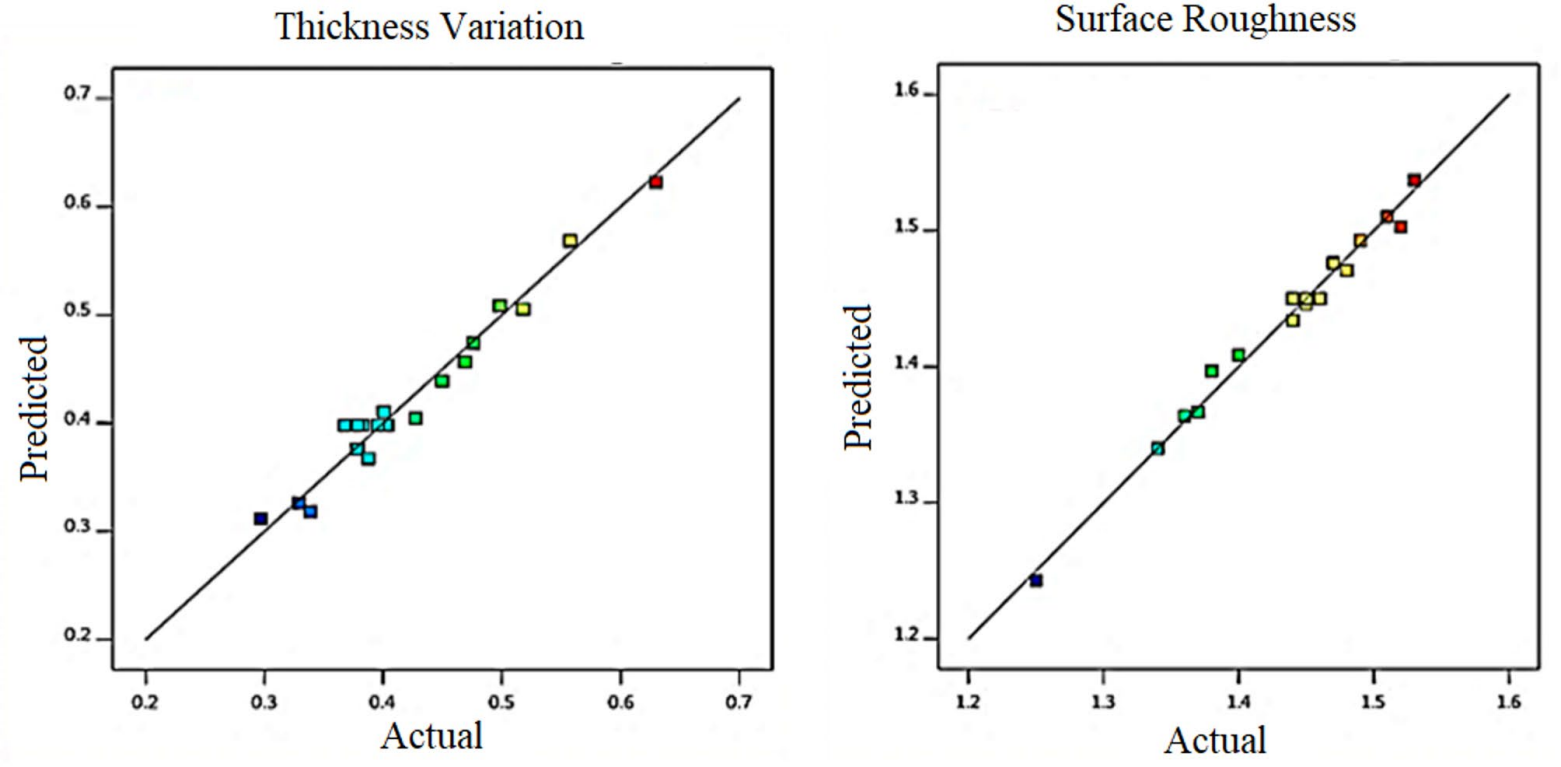
Correlation Between Actual and Predicted Values.

Figure 8 shows the correlation between the expected and experimental values. The actual and predicted values derived from the empirical model were compared to the output characteristics of the experimental samples. From the results, a better correlation was observed for each set of data. The R^2^ values of the generated empirical connection models fell within a region, which suggests a significant correlation between the actual and predicted values.

High-resolution SEM imaging was performed for visual confirmation of the coating quality. At an approximate magnification of 500x captured using the ThermoScientific Apreo S High-Resolution Scanning Electron Microscope. Examination of the SEM image further confirmed a uniform and continuous coating layer with minimal surface defects. The fine-grained structures and homogeneous texture indicated the success of the application and optimization of the spray parameters.

**Fig. 9:**
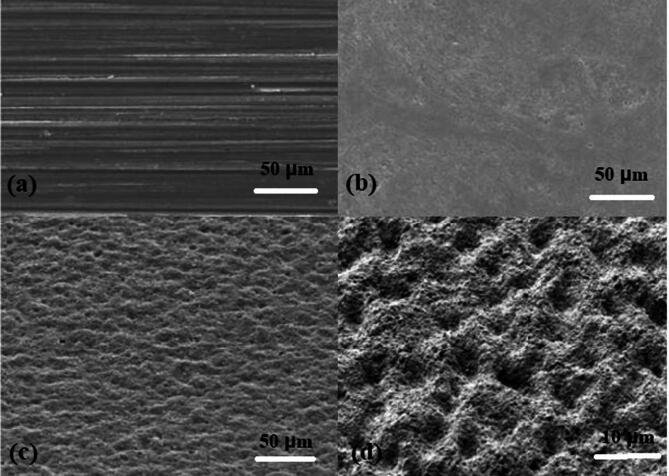
Surface morphology captured using SEM for Different Set Values.

Figure 9(a) polished/untreated surface showing parallel striations, Fig. 9 (b) mildly treated surface with slight roughening, Fig. 9 (c) moderately treated surface exhibiting uniform rough texture, and Fig. 9 (d) heavily treated surface with distinct porous microstructure at higher magnification. It showed good particle distribution and film formation in the absence of significant agglomerations or pinholes. Table 8 provides a detailed comparative analysis of the proposed system with similar studies. The average thickness and range of thickness in each case are mentioned along with their key findings as their remarks.

**Table 8:** Comparative analysis of the key findings from the previous literature.

References	Methodology	Average Thickness and Thickness Range (µm)	Remarks
^25^	Electrostatic Spray	0.60, (0.15–1.05)	Limited to conductive substrates, but good uniformity.
^26^	HVLP Spray	0.50, (0.10–0.80)	Less efficient in transferring paint but environmentally friendly.
^27^	Thermal Spray	0.55, (0.20–0.90)	Possesses a high deposition rate, but has the potential for thermal damage.
^28^	Deep Learning	0.53, (0.12–0.99)	Reduce over-spraying to enhance uniformity in coating but require large dataset.
^29^	Airless	0.57, (0.17–0.95)	Improved transfer efficiency but lacks in the optimization of nozzle.
Proposed work	Beta Distribution Model	0.58, (0.12–1.00)	Precise control of sub-micron coating thickness was achieved with optimized parameters and applicable to complex geometries.

**Fig. 10:**
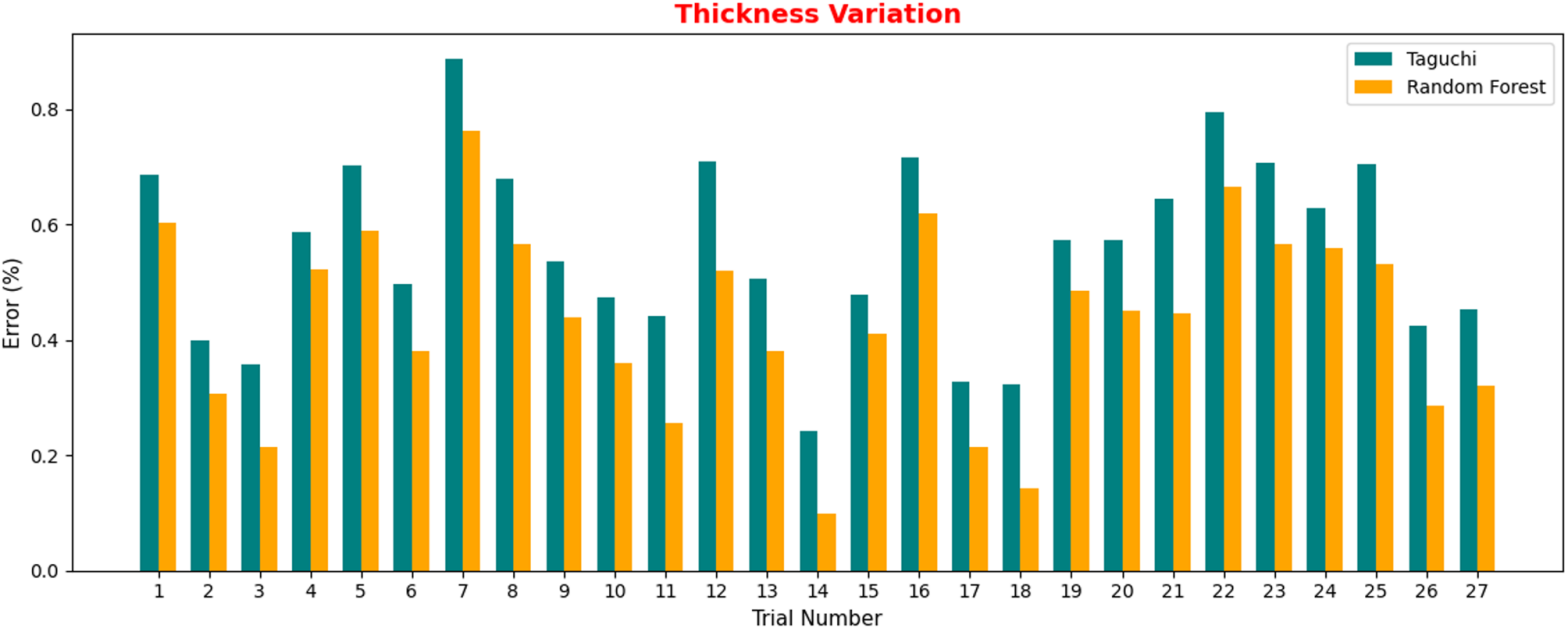
Validation for Thickness Variation.

Figure 10 shows the thickness variation analysis across eight trials and a comparison of the error from the Taguchi method and that from the Random Forest algorithm. The error percentage traverse was exhibited between trials, with the maximum error seen to be approximately 0.88% in trial 7 from the Taguchi method, and the Random Forest algorithm produced a comparatively lower error percentage, with a maximum error of approximately 0.76% in the same trial. Random Forest approach consistently resulted in lower and more reliable errors than the other approaches.

**Fig. 11:**
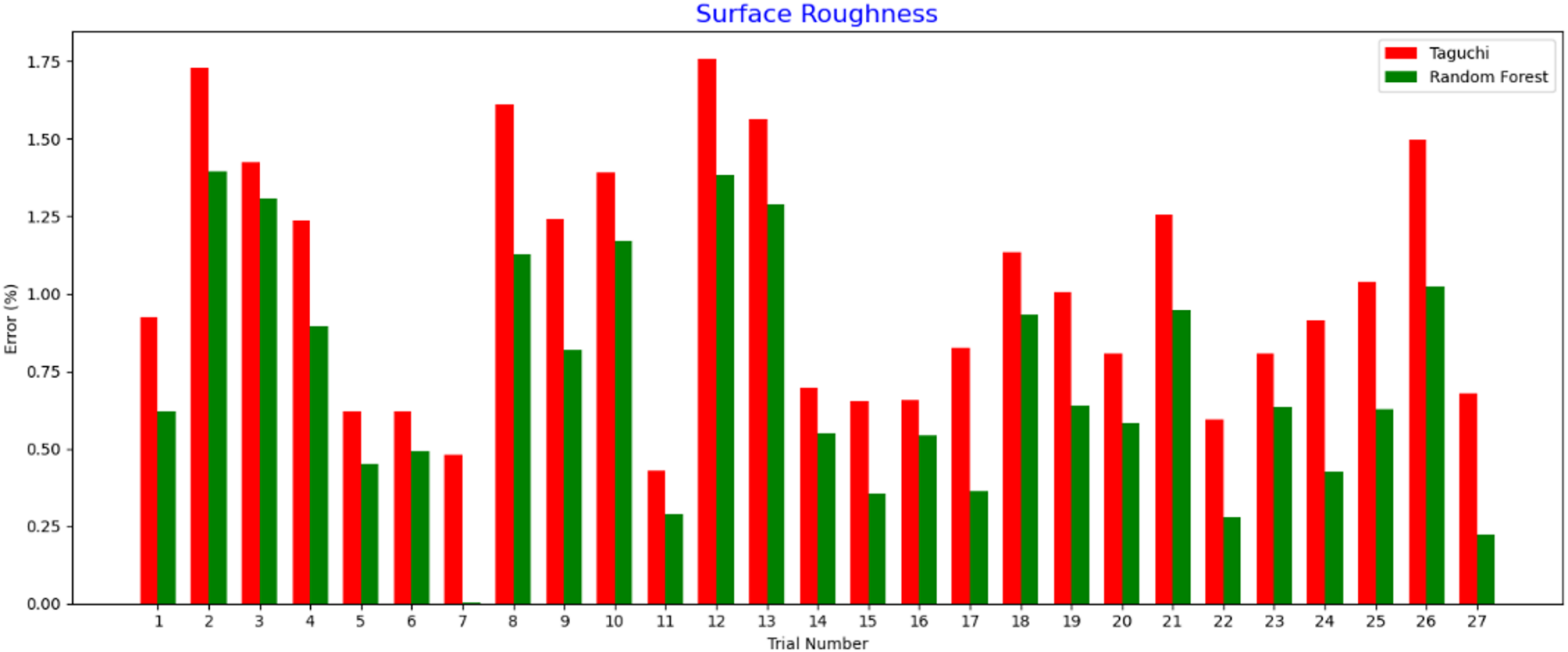
Validation for Surface Roughness.

As shown in Fig. 11, trials evaluating the surface roughness yielded error percentages. A maximum error of approximately 1.75% was obtained with the use of the Taguchi method in Trial 12, whereas a maximum error of approximately 1.49% was obtained for the Random Forest method. In particular, the surface roughness errors were smaller than the thickness variation in all trials, implying that both methods may predict the surface characteristics rather than the thickness parameters. Finally, owing to the consistency in Random Forest predictions it produced better surface quality and managed error rates in complex spray-painting processes.

Industrial robotic spray systems can use the proposed model for dynamic paint irregularity detection which enables the reduction of overspray and peel texture and inconsistent coatings. Industries can achieve more effective paint consumption while decreasing environmental impact and reducing costs through optimized spray setting optimization. Process control software that runs automatic robotic spray-painting systems can execute adaptive spray trajectory implementation through the application of this research study according to specific surface geometries. The developed findings enable manufacturers to make substrate-related viscosity and temperature modifications and set control parameters for spray velocity as well as establish predictive maintenance systems that detect and rectify manufacturing faults through machine learning-based technology. The predicted spray settings provide effective optimization, yet some important restrictions exist within the method. Only a single paint material was researched in this investigation which affects the predictive capabilities of the model when applied to other combinations of solutions and additives. Additional research needs to explore water-based coatings as well as high-viscosity enamels and metallic finishes.

## Conclusion

Automobile manufacturing heavily relies on precise paint applications through spray technology because this procedure determines product appearance together with operational service time. Manual spray painting addresses precise details, but industrial robots must become more productive for mass production because they provide exact operating capabilities. The research created a best-fit robotic spray trajectory model through implementing Taguchi Design of Experiments along with Random Forest regression to enhance coating uniformity control. Surface roughness and thickness variation evaluations under six control parameters used an L27 orthogonal array system to determine distance, pressure, temperature, humidity, speed and viscosity relationships. The experimental design allowed for three factor levels thus it provided an efficient way to uncover major parameters needing just a few experimental runs. The Taguchi method showed thickness variation depended mostly on viscosity and temperature parameters, yet speed and temperature became main factors influencing surface roughness. High delta values together with signal-to-noise ratio trends confirmed the research findings. Random Forest regression analysis linked up with physical experiments to understand the complicated relationships between numerous variables. R² values reached 0.9707 for thickness variation and 0.9224 for surface roughness while the predictive accuracy on this machine learning approach remained high. Tests on model validity showed prediction errors reaching a maximum of 0.76% for thickness while roughness predictions deviated by 1.49% which proved the model’s reliable operation. Together experimental design and machine learning served to analyze parameter sensitivities while creating accurate predictions. The design will advance through future work by adding extra factor levels and repeats and testing various paint substances and machine learning control programs within complex industrial spray environments.

## Data Availability

The datasets generated or analyzed during the current study are available from the corresponding author upon reasonable request.
